# Right-sided aortic arch with aberrant left subclavian artery diagnosed in an infant with regurgitation 

**Published:** 2022

**Authors:** Farid Imanzadeh, Amirhossein Hosseini, Mahsa Rashid, Ali Taher Ghasemi, Fatemeh Salahshouri, Fariba Fariba, Parin Yazdanifard

**Affiliations:** 1 *Pediatric Gastroenterology, Hepatology and Nutrition Research Center, Research Institute for Children’s Health, Shahid Beheshti University of Medical Sciences, Tehran, Iran*; 2 *School of Medicine, Shahid Beheshti University of Medical Sciences, Tehran, Iran*; 3 *Department Of Pediatric Cardiology, Mofid Children’s Hospital, Shahid Beheshti University of Medical Sciences, Tehran, Iran*

**Keywords:** Aortic Arch, Regurgitation, Infant

## Abstract

Right-sided aortic arch with aberrant left subclavian artery is a rare congenital anomaly of the aorta that occurs in less than 0.1% of the population. Patients are asymptomatic in most cases, and the anomaly is found incidentally; however, symptoms can occur due to the compression of other structures, mostly the trachea and esophagus. In this report, we present a case of esophageal compression by a right-sided aortic arch with aberrant left subclavian artery that mimicked gastro-esophageal reflux in a 3-month-old (87-day-old) infant with complaint of regurgitation, vomiting, and failure to gain weight who was diagnosed through a barium meal study.

## Introduction

 Infancy regurgitation and vomiting is a condition known to parents and physicians. Many such cases are not severe and are never referred for any further radiologic examinations, as their vomiting is usually related to gastro-esophageal reflux and is treated on that basis. However, severe or chronic cases are usually referred to pediatricians to perform further studies, which aim to identify any underlying pathologies and not to rule out gastro-esophageal reflux (1). These underlying disorders can be gastrointestinal abnormalities (e.g., gastric outlet obstruction, intermittent partial bowel obstruction, extrinsic compression of the GI tract, stenosis from ischemia, radiation, or Crohn’s disease, or achalasia) or non-gastroesophageal ones, like metabolic abnormalities (e.g., metabolic acidosis as seen in food protein-induced enterocolitis syndrome and urea cycle disorders), or any other underlying conditions, like abnormal compression effects from neighboring organs like thoracic vascular rings (2, 3). Vascular rings, which happen due to anomalies in major arteries like the aortic artery and its branches, can put pressure on the trachea or esophagus and lead to respiratory distress and dysphagia (4-7). A right-sided aortic arch with aberrant left subclavian is one of these anomalies with rare occurrence ranging from 0.05% to 0.1% of the population. Most cases are asymptomatic; however, some show esophageal or respiratory compression symptoms (4-10). Herein, we report a case of esophageal compression by a right-sided aortic arch with aberrant left subclavian that mimicked gastro-esophageal reflux in an 87-day-old infant with complaint of regurgitation, vomiting, and failure to gain weight who was diagnosed by a barium study.

## Case report

**Figure 1:**
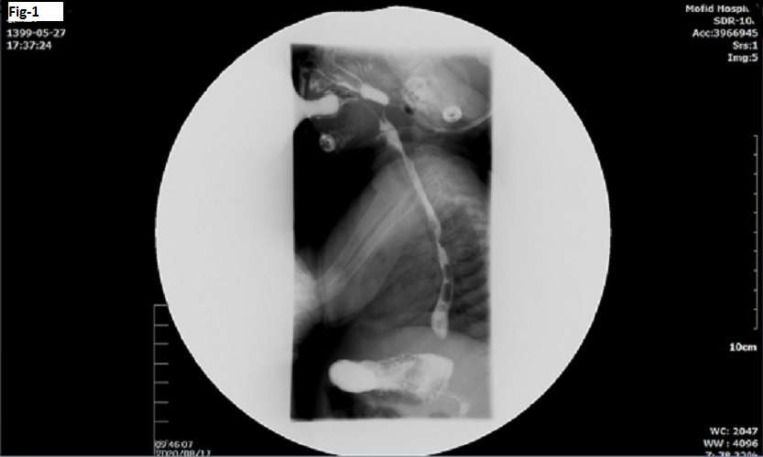
Upper gastrointestinal series; arrow shows compression effect on posterior aspect of esophagus at the level of aorta arc suggestive of aberrant right subclavian artery

An 87-day-old female infant presented to the hospital with increasingly frequent non-bilious vomiting and regurgitation in addition to failure to gain weight and weight loss according to her mother. She was a well-baby with no diseases or anomalies and weighed 2.7 kg at birth. During the last 20 days prior to admission, she experienced frequent and increasing regurgitation and vomiting, causing the loss of 400 grams more of her weight. At the time of admission, the patient weighed 4.5 kg with a Z-score of -2 (weight for length). She was afebrile with stable vital signs for her age, including a pulse rate of 120 beats/min, a respiratory rate of 23 breaths/min, and blood pressure of 90/60 (mm Hg). Initial physical examination revealed nothing but failure to thrive. She was neither ill, nor toxic, nor cyanotic. Her heart sounds were normal and rhythmic without any murmurs. Respiratory sounds were clear. The abdomen was soft with active bowel sounds. The rest of her physical examination and electrocardiogram (ECG) were also normal. All laboratory findings including complete blood count, urine, and stool exams were normal. The abdominal ultrasound revealed stomach contents passing through a non-hypertrophied pylorus that was seen to open and close normally. A small amount of free fluids was detected in the Morrison space with a spleen size of 61 mm on midclavicular line. Pelvic and prominent mesenteric lymph nodes were estimated to be up to 4 mm. The infant’s upper gastrointestinal (GI) series showed nasopharyngeal regurgitation and compression effect on posterior aspect of esophagus at the level of aorta arc, which was suggestive of double aortic arch or, less probably, aberrant right subclavian artery (Figure 1). A barium meal study revealed severe gastroesophageal reflux, and her small bowel follow through revealed a nodular filling defect in the ileac loops, suggestive of nodular lymphoid hyperplasia. Echocardiogram, which was highly recommended due to her barium study results, showed a right-sided aortic arch with a small atrial septal defect (ASD). Cardiac function and pulmonary artery pressure (PAP) were reported to be normal. 

**Figure 2:**
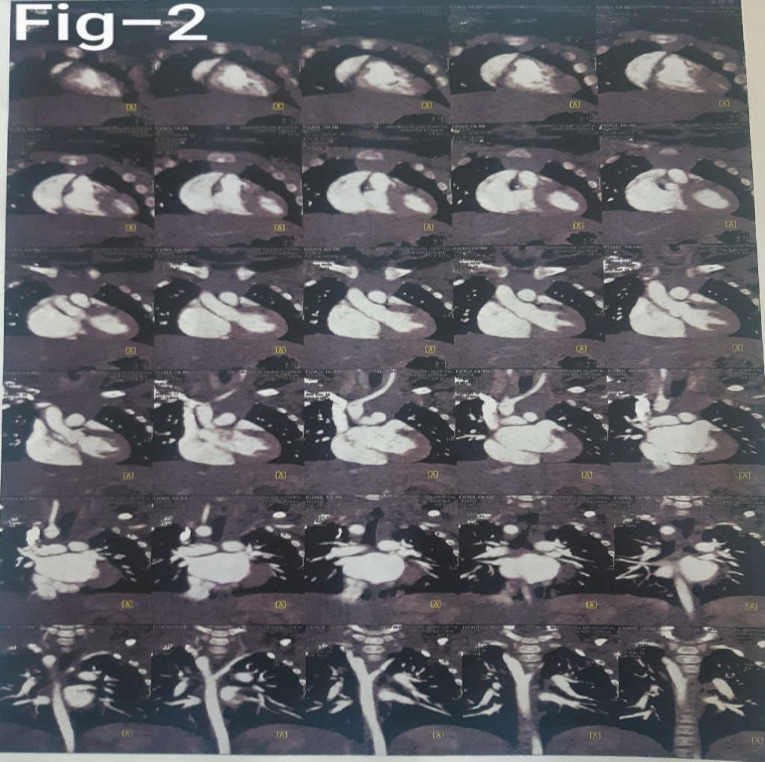
Multi-slice spiral computerized tomography (CT) angiography; Right-sided aortic arch with an aberrant left subclavian artery with notable compression on esophagus and prominent main pulmonary artery and its branches due to left to right shunt

The patient underwent a multi-slice spiral computerized tomography (CT) angiography of the heart and vasculature using a non-ionic contrast and a saline chaser via dual injector through the intravenous route. Multiple axial and sagittal images with multi-planar reformation (MPR), maximum intensity projection (MIP), and volume rendering technique (VRT) reconstruction showed a SDS levo-cardiac heart with atrioventricular and ventriculoarterial concordance and intact ventricular septum (IVS) with no sign of permeant ductus arteriosus (PDA). It also revealed a right-sided aortic arch with an aberrant left subclavian artery with notable compression on the esophagus. The main pulmonary artery and its branches were prominent due to a left-to-right shunt. All pulmonary veins returned to the left atrium with no sign of left superior vena cava (LSVC) (Figure 2).

## Discussion

Diagnosis and treatment of major arterial anomalies were previously established. In 1794, Byford described an abnormal right subclavian artery which was found during the autopsy of a woman who died of starvation due to this anomaly. Furthermore, in 1837, von Sibold described a double aortic arch. Later, in 1945, a double aortic arch was found in a 4-month-old infant. This discovery led to further studies finalizing and categorizing these vascular anomalies (4).

In early embryologic stages, the aortic arches start off as a duplicate system. Later, the right aortic arch turns into the right subclavian and common carotid artery. An aberrant right subclavian artery is the outcome of an abnormal involution of the right aortic arch with a persistent intersegmental artery. This aberrant subclavian, or lusoria, artery goes through the mediastinum between the esophagus and the vertebral column to reach the right axilla in the majority of cases and can cause pressure on nearby organs such as the esophagus and trachea (4-10). Obviously, clinical presentation of vascular rings and anomalies are different by their types and effects on nearby structures. Patients with double aortic arch often have apparent clinical signs, although those with aberrant subclavian artery are rarely symptomatic. Major clinical pictures of these vascular rings are mostly respiratory embarrassment, distress, or recurrent respiratory tract infections followed by dysphagia, poor feeding, and other gastroesophageal signs (4-11). This is possibly because of the lack of tracheal rigidity, allowing its compression to obstruct the airway and cause recurrent pulmonary infections. Increasing rigidity of the esophagus or arterial elongation and thickening due to atherosclerosis are considered to be the causes of dysphagia in older patients. Another theory is the coincidence of a common carotid origin and an aberrant right subclavian artery can give rise to compression of the esophagus between these vessels (4-6, 11).

In some research, the barium swallow test has been indicated as the most accurate and sometimes the only way to diagnose these types of anomalies. In the current case, a barium swallow study was indicated and showed narrowing of the esophagus on both anterior and posterior sides due to pressure of aortic arch, which caused apparent pulsating indentations. These indentations signs are very rare; however, they can be seen during an endoscopy. CT-angiography and MRI-angiography (magnetic resonance imaging) are other imaging studies also considered as the gold standard for diagnosing right subclavian artery abnormalities. Plain chest radiographs can be helpful only in diagnosing right-sided aortic arch with left ducted ligamentum (4, 5).

As seen in the current case, despite all major vascular anomalies which are usually life threatening and need serious clinical interpretations, right-sided aortic arch with aberrant left subclavian artery can be found accidentally during further assessments of infants with not serious but chronic and non-responsive respiratory and esophageal symptoms.

## Conflict of interests

The authors declare that they have no conflict of interest.
